# Highly accurate, automated quantification of 2D/3D orientation for cerebrovasculature using window optimizing method

**DOI:** 10.1117/1.JBO.27.10.105003

**Published:** 2022-10-22

**Authors:** Jia Meng, Lingxi Zhou, Shuhao Qian, Chuncheng Wang, Zhe Feng, Shenyi Jiang, Rushan Jiang, Zhihua Ding, Jun Qian, Shuangmu Zhuo, Zhiyi Liu

**Affiliations:** a Zhejiang University, College of Optical Science and Engineering, International Research Center for Advanced Photonics, State Key Laboratory of Modern Optical Instrumentation, Hangzhou, China; b Zhejiang University, Jiaxing Research Institute, Intelligent Optics & Photonics Research Center, Jiaxing, China; c Jimei University, School of Science, Xiamen, China

**Keywords:** cerebrovasculature, orientation, window optimizing, aggregation-induced emission

## Abstract

**Significance:**

Deep-imaging of cerebral vessels and accurate organizational characterization are vital to understanding the relationship between tissue structure and function.

**Aim:**

We aim at large-depth imaging of the mouse brain vessels based on aggregation-induced emission luminogens (AIEgens), and we create a new algorithm to characterize the spatial orientation adaptively with superior accuracy.

**Approach:**

Assisted by AIEgens with near-infrared-II excitation, three-photon fluorescence (3PF) images of large-depth cerebral blood vessels are captured. A window optimizing (WO) method is developed for highly accurate, automated 2D/3D orientation determination. The application of this system is demonstrated by establishing the orientational architecture of mouse cerebrovasculature down to the millimeter-level depth.

**Results:**

The WO method is proved to have significantly higher accuracy in both 2D and 3D cases than the method with a fixed window size. Depth- and diameter-dependent orientation information is acquired based on *in vivo* 3PF imaging and the WO analysis of cerebral vessel images with a penetration depth of 800  μm in mice.

**Conclusions:**

We built an imaging and analysis system for cerebrovasculature that is conducive to applications in neuroscience and clinical fields.

## 1 Introduction

Cerebral vessel imaging and analysis are vital to neuroscience and related clinical applications. The remodeling of blood vessels is closely associated with disease evolution, wound healing, and development of tissues.^1^^,^^2^ Before pathological hallmarks of Alzheimer’s disease arise, changes appear in the vascular morphology.^3^ Blood vessel alterations, blood–brain barrier disruption, and cerebral blood flow abnormalities are also described in amyotrophic lateral sclerosis and Parkinson’s disease.^4^^–^^6^ Therefore, large-depth imaging of cerebral vessels and quantitative characterization techniques to resolve subtle morphological changes enable a better understanding of the relationship between tissue structure and function.^7^

For deep-penetration brain imaging, although x-ray computed tomography and magnetic resonance imaging have been widely utilized, they have some limitations due to the relatively low spatial resolution.^8^^,^^9^ Excellent reliability and biocompatibility make aggregation-induced emission (AIE) dots great candidates for fluorescent biomedical imaging.^10^ However, the photon absorption and scattering of excitation or emission light influence its penetration depth. Owing to reduced absorption and scattering, AIE based on the second near-infrared (NIR-II) region for multi-photon fluorescence imaging is quite promising for observing large-depth brain structures.

Spatial orientation is one of the most important vessel features; it serves as an indicator for diagnosing diseases, locating injuries, and evaluating tissue development. It is also the basis for defining the alignment of fibrous structures.^11^^,^^12^ Previous methods typically obtained the mean orientation of an image or a region of interest, such as the techniques relying on Fourier transform^13^^,^^14^ or Hough transform.^15^ Bancelin et al.^16^ proposed a morphological open operation method to realize visual spatial orientation, but it was only applicable to the case of similar fiber diameters. Quinn and Georgakoudi proposed a weighted orientation vector summation algorithm that was able to acquire pixel-wise orientation for 2D images,^17^ and Liu et al. further extended this method to 3D forms.^18^ The 2D/3D weighted vector summation algorithm assumed that morphological features of fibrous structures were identical and employed a fixed window size for all fibers within a 2D/3D image, with the optimal window size being 2 to 4 times the fiber diameter.^17^^,^^18^ Therefore, these methods might suffer from the degradation in orientation determination accuracy when applied to complex systems with varying fibrous thickness, such as cerebrovasculature.

Here, we built a system for large-depth imaging of cerebral vessels and adaptive analysis of orientation. Specially designed AIE nanoparticles (NPs) were used to obtain large-depth 3D cerebrovascular image information. Recently, we developed an automated, voxel-wise measure of thickness within fiber-like structures and applied it to the analysis of cerebrovascular diseases.^19^ Based on the thickness information, in this study we propose a window optimizing (WO) method that is able to significantly enhance the determination accuracy of spatial orientation, for both 2D and 3D cases. As a fusion of thickness determination and a weighted orientation vector summation algorithm, the WO method adaptively optimizes calculating parameters at a pixel-wise basis according to fiber thickness information. We assess the performance of this method through simulated 2D and 3D fiber images. Finally, we demonstrate the application of this system by establishing the orientational architecture of large-depth 3D images of mouse cerebrovasculature acquired from AIE-assisted *in vivo* three-photon fluorescence (3PF) imaging.

## 2 Experimental Set-Up

Specially designed AIE NPs, called DCDPP-2TPA, were used to obtain large-depth 3D cerebrovascular images. The reported DCDPP NPs showed strong electron-accepting ability.^20^ To obtain luminogens with distinct multiphoton absorption capability, we modified the DCDPP with TPA, which was a strong electron donor, to construct the donor-acceptor structure. DCDPP-2TPA had a large three-photon-absorption cross section at 1550 nm and deep-red emission, which was suitable for 3PF imaging.^21^ The 3PF spectrum of DCDPP-2TPA is shown in Fig. S1 in the Supplementary Material.

**Fig. 1:**
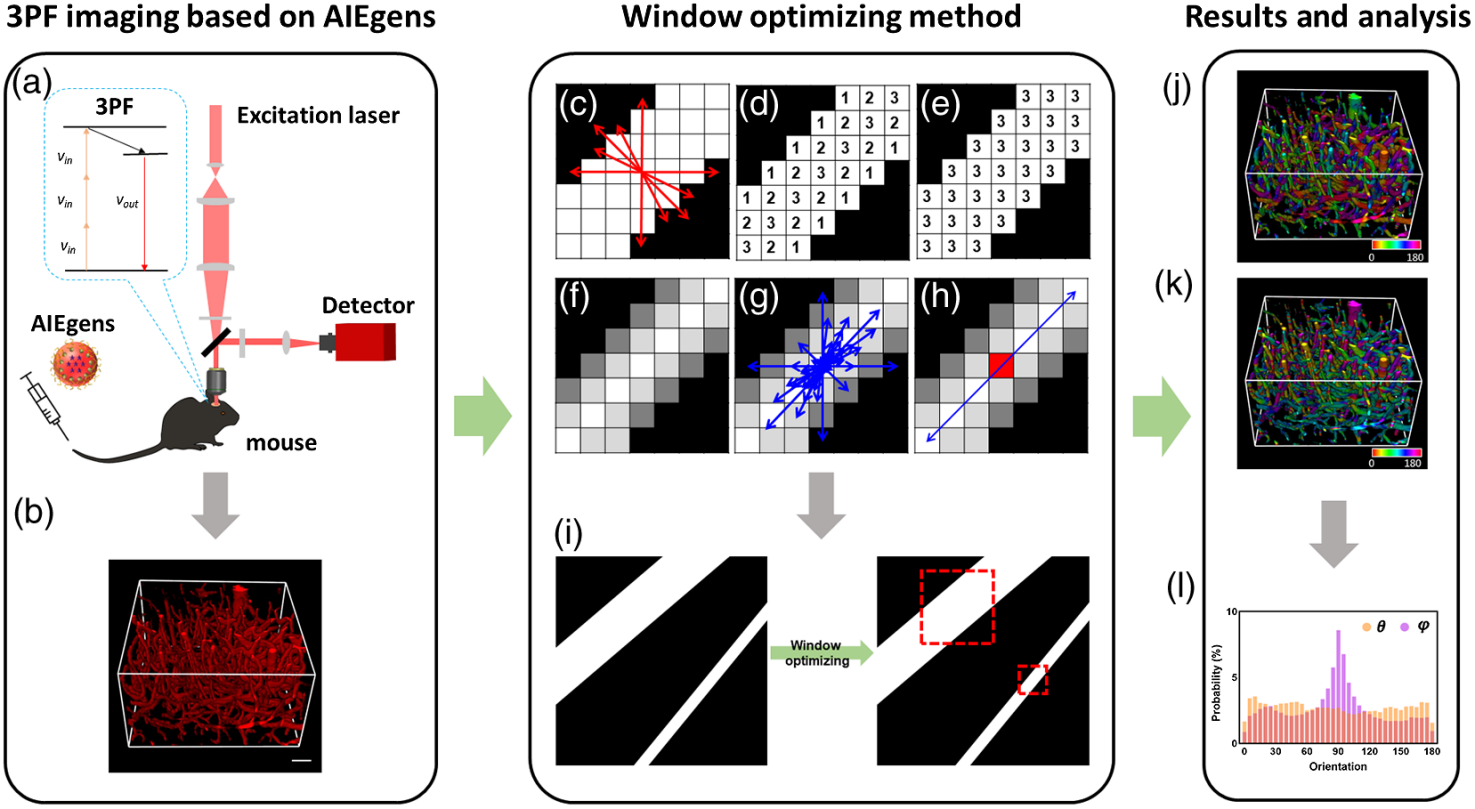
Schematic diagram of imaging and characterizing system. (a) NIR-II region imaging system based on aggregation-induced emission luminogens. (b) 3D reconstruction of mice brain blood vessels. (c) Minimal distance searching. (d) Distance transfer within the fiber image. (e) Pixel-wise thickness results. (f)–(h) Weighted orientation vector summation algorithm for orientation calculation. (i) Adaptive window size generation based on thickness information. (j) θ and (k) φ orientation maps of the 3D vessel image stack. (l) Orientation distribution histograms.

The mice experimental procedures were approved by the Animal Use and Care Committee at Zhejiang University (ZJU20190076) and in accordance with the National Institutes of Health Guidelines. Female BALB/c mice used in the experiment were located at a room temperature of ∼24°C with a 12-h light/dark cycle and were fed with standard chow and water. Mice skulls were opened through microsurgery. A cover glass slide was mounted onto the opened brain to offer a cranial window. Each mouse was intravenously injected with DCDPP-2TPA NPs. After that, an upright scanning microscope (Olympus, BX61W1-FV1200) with a laser source (central wavelength of 1550 nm, pulse width of 400 fs, and repetition frequency of 1 MHz) was used for *in vivo* 3PF imaging. A water-immersion objective lens (XLPlan N, 25×, NA 1.05, work distance = 2.0 mm) was used to focus the laser beam on the mice. The 3PF signals filtered by a 650-nm long-pass filter were collected by PMT. The schematic diagrams of the 3PF imaging system and 3D cerebrovascular image stack are shown in Figs. 1(a) and 1(b). The lateral resolution of the system was 0.83  μm, and the axial resolution was 2.75  μm. A total of four mice were used for imaging to establish the orientational architecture of brain blood vessels.

## 3 Results

### 3.1 Pixel-Wise Vessel Orientation Characterization Method and Accuracy Test

When calculating the pixel-level morphological features of fibrous structures (such as blood vessels), it was usually necessary to set the window size and obtain the eigenvalues of the central pixel. Generally, the window size is related to the diameter (thickness) of fibers. When using our previously proposed weighted orientation vector summation algorithm, we estimated the fiber diameter for the determination of window size and typically used the fixed window size for each pixel of an image. However, it might lead to undersampling (the window size is too small) or neighborhood contamination (the window size is too large), which would degrade the determination accuracy of orientation. In contrast, with the WO method, we assessed the fiber diameter at each pixel for the optimal window size at a pixel-wise basis. Specifically, we started from obtaining fiber diameter information, relying on the adaptive distance transmission approach [Figs. 1(c)–1(e)], as detailed in Fig. S2 in the Supplementary Material. The accuracy of the thickness assessment was tested, and a superb accuracy with an error level below 1% was achieved (Fig. S3 in the Supplementary Material). Then, the window size of each pixel was determined automatically and was usually set to 2d+1, where d is the thickness value of each pixel/voxel [Fig. 1(g)]. The weighted orientation vector summation algorithm [Figs. 1(f)–1(h) and Fig. S4 in the Supplementary Material] was then employed to acquire 2D/3D orientations relying on optimized window size, with red dashed boxes representing suitable ones for fibers with different diameters [Fig. 1(i)]. In 2D space, a certain orientation could be described by the azimuthal angle θ, whereas both the azimuthal angle θ and the polar angle φ were needed to describe an orientation in 3D space (Fig. S5 in the Supplementary Material), with θ and φ ranging from 0 deg to 180 deg. Finally, we obtained orientation maps at a pixel-wise basis [Figs. 1(j) and (1k)] and acquired their distribution features [Fig. 1(l)].

**Fig. 2:**
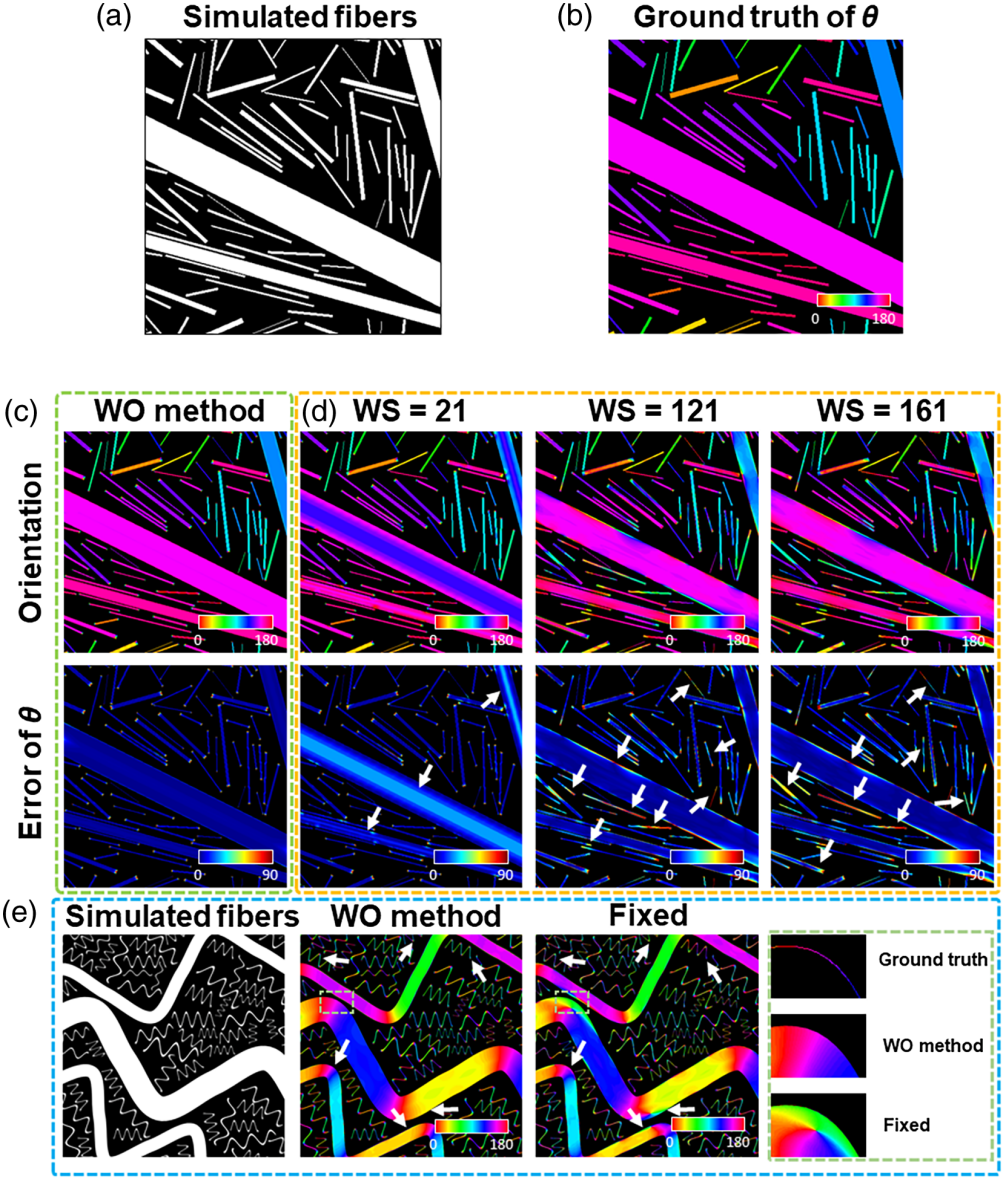
Performance of the WO method in characterizing 2D images. (a) Simulated fiber image. (b) Ground truth of the fiber orientation. (c) Orientation (top) and error (bottom) maps from the WO method. (d) Orientation (top) and error (bottom) maps from the fixed-window method based on different window sizes. (e) Simulated curvy fibers (left), along with orientation maps obtained from the WO (middle) and the fixed-window method with a window size of 81 pixels (right). The marked region (by dashed green box) is zoomed in to compare the acquired orientation outputs from the WO and the fixed-window method. White arrows point to local regions with inaccurate orientation results in (d) and ROIs where a more accurate orientation is achived from the WO method in (e).

To evaluate the accuracy of this algorithm, we first tested it on 2D images (each with a size of 500×500  pixels) of simulated fibers with more than 10 different diameters of 1 to 60 pixels [Fig. 2(a)]. The orientation information of these fibers was acquired simultaneously and served as the ground truth [Fig. 2(b)]. Then, we compared the WO method [Fig. 2(c)] with the fixed-window ones [Fig. 2(d)] in the performance of orientation determination, which can be clearly observed from the corresponding error maps. As can be seen, the orientation results achieved from the WO method were very close to the ground truth, with a low level in error [Fig. 2(c)]. However, the method with the fixed window sizes led to a relatively higher level of θ error. The conventional algorithm accurately characterized thinner fibers when the window size was relatively small, but obvious calculation errors occurred near the central area of thicker fibers. By contrast, when the fixed window size increased, it worked better for thicker fibers, but the calculated results for finer ones became worse [Fig. 2(d)]. For fiber images with a diameter of 1 to 60 pixels and 10 distinct sizes, we acquired the orientation error using the WO method and the fixed-window method with varying window sizes ranging from 21 to 201 pixels (Fig. S6 in the Supplementary Material). We found that the average angle error from the WO method was ∼3  deg, whereas that from the fixed-window method was typically higher than ∼9  deg (n=4 images, as detailed in Fig. S6). In addition to the images with straight fibers, the WO method performed better than the window-fixed one for images with simulated curvy fibers (500×500  pixels in size), ranging 4 to 60 pixels in diameter [Fig. 2(e)]. We note that the maps shown in Fig. 2(e) were orientation maps not error maps. In particular, we extracted the boundary of a curved part of a fiber and obtained its true orientation. The orientation map acquired from the WO method was very close to the ground truth, whereas obvious differences (a piece of green-colored area) were observed from the orientation map obtained using the fixed-window method [insets, Fig. 2(e)]. These results reveal that the fixed-window method might have difficulty accurately characterizing complex biological systems typical of varying fiber thickness, whereas the WO method significantly improved the calculation accuracy by adaptively determining the optimized window size (with quantitative comparison results shown in Fig. S6 in the Supplementary Material).

**Fig. 3:**
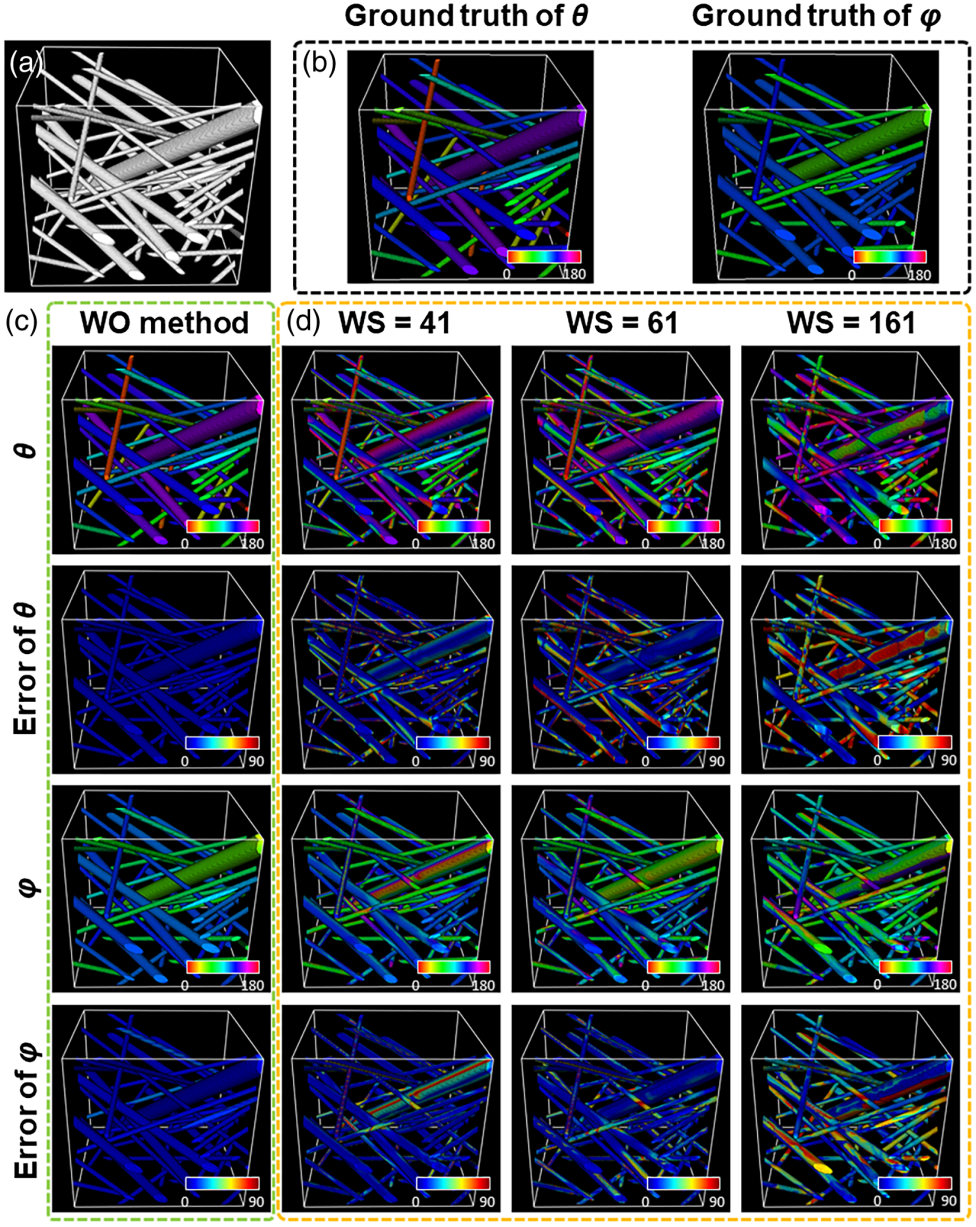
Performance of the WO method in characterizing 3D images. (a) A representative simulated 3D fiber stack. (b) The ground truth of θ and φ orientation maps. (c) Orientation and error maps from the WO method. (d) Orientation and error maps from the fixed-window method based on different window sizes.

Similarly, we assessed the performance of the WO method in the 3D case. Simulated 3D fiber stacks (each with a size of 300×300×300  voxels) with varying diameters of 8 to 30 voxels (including three distinct diamaters at 8, 15, and 30 voxels, respectively) were tested [Fig. 3(a)]. The ground truth of θ and φ orientation maps is shown in Fig. 3(b). We then used the WO method and the method with fixed window size to calculate the orientation of 3D fibers, respectively. As can be seen from the calculated orientation and error maps, the WO method led to highly consistent orientation results with the ground truth, as visualized from a very low level of errors for both angles [Fig. 3(c)]. However, analysis results achieved from the fixed-window method with varying window sizes were not comparable to that from the WO method, with detailed error levels at different window sizes shown in Fig. S7 in the Supplementary Material. In addition to the error levels of θ [Fig. S7(a) in the Supplementary Material] and φ [Fig. S7(b) in the Supplementary Material], we also acquired the angle between the real and the calculated orientation in the 3D case [Figs. S7(c) and S7(d) in the Supplementary Material].The error levels acquired from window sizes close to the true fiber diameters (i.e., 8 to 30 voxels) are shown in the inset of Figs. S7(a), S7(b), and S7(d) in the Supplementary Material. These results indicate that the WO method consistently performed better than the fixed-window method with the window size varying from 7 to 201 voxels. According to quantitative readouts (Fig. S7 in the Supplementary Material), we prepared the orientation maps and corresponding error maps at the fixed window size of 41, 61, and 161 voxels [Fig. 3(d)], where the window sizes of 41 voxels and 61 voxels were almost the optimized ones (corresponding to the lowest error levels) for the simulated 3D image, and the window with a size of 161 voxels was able to provide the estimation of error levels in the oversized window case. Similar to the 2D case, when the window size was small, the error level of thick fibers became obvious, and the increased window size caused a degradation in the orientation accuracy mainly for thin fibers [Fig. 3(d)]. These results reveal that the WO method leads to significantly improved orientation determination accuracy in the 3D case as well.

### 3.2 3D Organizational Analysis of Cerebral Vessels in Living Mouse

**Fig. 4:**
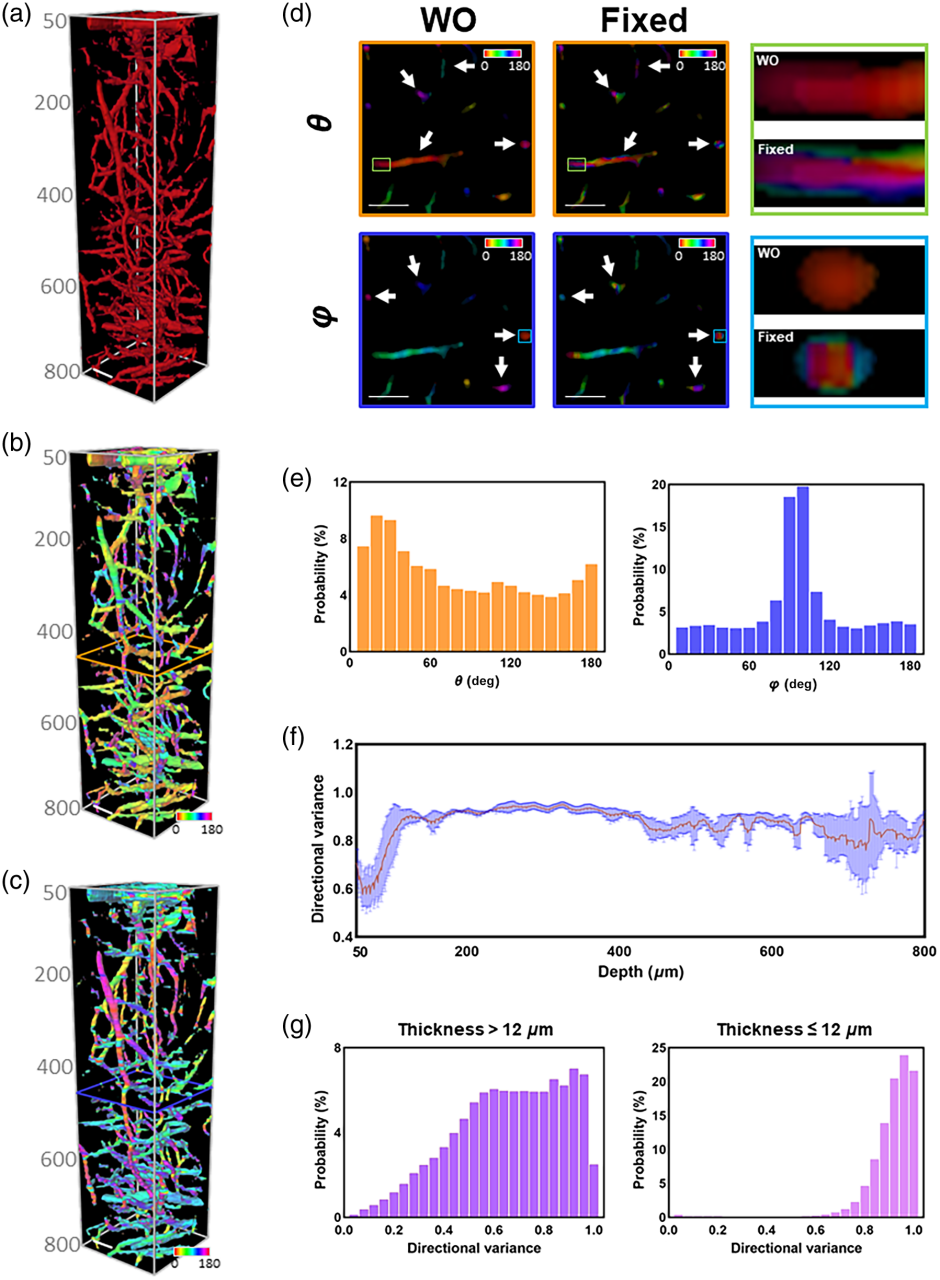
3D images of mouse cerebral blood vessels with corresponding orientation analysis results acquired from the WO method. (a) 3D 3PF images of brain blood vessels from mice. Scale bar: 50  μm. Corresponding θ (b) and φ (c) orientation maps of the blood vessel stack. (d) Comparison of the WO method and the fixed-window method. Scale bar: 50  μm. (e) Probability distribution of θ and φ assessed from the entire 3D stack. (f) Depth-dependent profile of directional variance of mouse brain blood vessels (n=4 mice). (g) Probability distribution of the directional variance of blood vessels with different thickness levels.

To assess the applicability of this imaging and characterizing platform, we obtained 3D fluorescence images of cerebral blood vessels from mice assisted with DCDPP-2TPA particles. The 3PF images of mouse brain blood vessels are shown in Fig. 4(a). The penetration depth was as large as 800  μm. These blood vessels in the field of view had a large density and an extensive range of diameters [Fig. 4(a)], forming an extremely complex system that was particularly suitable as the object of the WO method. We obtained corresponding θ [Fig. 4(b)] and φ [Fig. 4(c)] orientation maps using the proposed WO method. For comparison, we used the fixed-window method to analyze these blood vessels. The representative maps from the WO and fixed-window methods are shown at the depth of 430  μm [Fig. 4(d)]. It is worth noting that the maps shown in Fig. 4(d) were orientation maps not error maps. As can be seen from the zoomed-in regions, there were generally uniform hues within a certain vessel as resolved from the WO method [Fig. 4(d)], which corresponded well to the real situation. However, the fixed-window method led to obvious inconsistent hues (i.e., distinct orientations) even within the cross-section of a certain vessel [Fig. 4(d)]. These observations reveal an improvement in determination accuracy by the WO method in the context of real biological samples. Through the quantification of 3D blood vessels, we were able to obtain the orientation distribution of the cerebrovasculature [Fig. 4(e)]. A higher level of φ near 90 deg reveals that most blood vessels were parallel to the imaging plane, whereas the relatively uniform distribution of θ indicates randomly orientated vessel distribution [Fig. 4(e)]. In addition, we generated 2D vessel image by projecting 3D images within a certain depth range and showed an improvement in orientation determination accuracy using the 2D WO method compared with the fixed-window one as well (Fig. S8 in the Supplementary Material).

Based on the 3D orientation map, we calculated the 3D directional variance of blood vessels to represent the alignment level (with methods detailed in in the Supplementary Material). Typically, when the depth was <120  μm, mouse brain blood vessels aligned orderly (corresponding to relatively low variance level), whereas more randomly oriented capillaries dominated the region with a depth >120  μm [Fig. 4(f)]. To validate these observations, we analyzed the blood vessels with different thicknesses separately [Fig. 4(g)]. When the thickness was <12  μm, the capillaries showed a high variance level, which meant that the arrangement of blood vessels was more disordered. In contrast, the remaining thicker blood vessels were relatively more aligned. Therefore, the WO method not only obtained more accurate spatial orientation, but also provided information of vessels with different diameters separately, owing to quantitative characterization at a voxel-wise basis.

## 4 Discussion and Conclusion

In this study, we proposed the WO method, which is a fusion of the thickness determination and weighted vector summation algorithm, both of which were developed in our previous studies. An important characteristic of the thickness algorithm is its ability to provide pixel-wise thickness information, in contrast to the majority of established methods. Some of these methods led to numerical results that cannot be used for visual purpose.^22^ Some methods were able to provide the visual presentation of vessel thickness, but they needed to skeletonize blood vessels, which might destroy the original morphological information.^23^ Owing to the ability of offering pixel-wise thickness information, in the WO method, we were able to optimize the window size at each pixel, which contributes to the enhancement of accuracy in orientation determination.

Assisted by the pixel-wise thickness information, the proposed WO method has shown good performance both in simulation and experiment. Due to the additional diameter evaluation process, the WO method tends to take a relatively longer time to quantify the spatial orientation compared with the fixed-window method. Taking the image in Fig. 2 as an example, the computational time required by the WO method was approximately 65 s on a desktop computer with an AMD Ryzen 5 3600X processor (3.79 GHz) and 16 GB of RAM, whereas the time required by the fixed-window method with a window size of 121×121  pixels and exactly the same computer configuration was 45 s. This is a trade-off between computation time and accuracy. In many cases, it is worthwhile to spend relatively more time in exchange for automated quantification and a higher level of accuracy.

Most of the reported methods are not able to provide pixel-wise characterizations of orientation; these include the FIRE, CT-FIRE, FiberFit, and Fourier transform methods.^24^^–^^26^ Although they are independent of the window size, they only yield average readouts of the entire image or a certain region of interest (ROI). Figure S9 in the Supplementary Material shows the analysis results of the FiberFit method, and the acquired average orientation of the entire image is compared with the true value and the one acquired from the WO method [Fig. S9(d) in the Supplementary Material]. As can be seen from the comparison, the FiberFit method does not perform well for the image with large variations in fiber diameters. Some other methods are able to provide the pixel-wise readouts, such as the one using the morphological opening.^16^ A structuring element (Strel) is utilized to extract fibers in the Strel direction, which needs to be determinated according to the size of fibers before the calculation. Letting the Strel rotate, a collection of openings by this rotatable line results in a stack of images, and the local orientation of each pixel of the image could be obtained accordingly. This method does not require setting the window size, but it does need to set a fixed proper Strel length. Therefore, it is well suited for calculations under diameter approximation conditions, as shown in Figs. S10(a)–S10(c) in the Supplementary Material. However, serious errors occur under conditions of large diameter differences [Figs. S10(d)–S10(f) in the Supplementary Material]; such errors could be reduced by the WO method.

In conclusion, we developed a quantitative imaging and characterizing system aiming to observe large-depth brain blood vessel structures and resolve spatial orientation with biological adaptability. We use specially designed AIE particles to achieve 3PF imaging of mice cerebral vessels with the penetration depth of 800  μm. A new technique called the WO algorithm was proposed; to enable optimizing the window size for orientation calculation adaptively. This method is verified to have significantly higher accuracy in both 2D and 3D cases than the method with a fixed window size. We anticipate that this system will have broad application prospects in precision medicine as a result of the highly-accurate characterization of cerebral organization alterations for neuroscience and related clinical applications.
