# Supplementary material for: Highly accurate, automated quantification of 2D/3D orientation for cerebrovasculature using window optimizing method
Source: J Biomed Opt. 2022 Oct 22;27(10):105003. doi: 10.1117/1.JBO.27.10.105003 (PMC9587757; doi:10.1117/1.JBO.27.10.105003)
Supplement: Supplementary file 1 [file JBO_027_105003_SD001.pdf]

Highly-accurate, automated quantification of 2D/3D orientation for cerebrovasculature using window optimizing method method

1. Supplementary Methods

1.1 Assessing the performance of thickness algorithm

The WO method proposed in this paper was based on the pixel/voxel-wise thickness determination. We tested the accuracy of the thickness algorithm using simulated fibrillar structures (Fig. S3). Fiber stacks were generated with varying diameters of 8-15 pixels, with a sample size of 8 stacks at each distinct diameter. The relationship between the defined fiber diameter and the calculated thickness was acquired, along with corresponding determination error level.

1.2 Orientation quantification of 2D cerebral vessels based on WO method

Intraoperative imaging generally led to 2D images. Therefore, quantitative characterization of 2D cerebral vessels was of great significance. Herein, we projected a certain depth of 3D blood vessels onto a 2D plane, and compared the orientation determination accuracy obtained from the WO method and the fixed-window one (Fig. S8).

1.3 Description of 3D directional variance

The 3D directional variance was a metric based on voxel-wise orientation information to measure the alignment of fibers in 3D space[11]. Here we consider the additional angles β and γ to obtain fixed 2D planes which fibers could be projected to for the determination of all possible φ orientations, as shown in Fig. S5(c). The determination of β and γ could be achieved using the same approach as that for θ. 3D directional variance ranged between 0 and 1, with 0 corresponding to perfectly parallel alignment, while 1 corresponding to complete randomness. The directional variance was defined as:

$$Variance_{3D} = 1 - R_{mean} \tag{1}$$

where $R_{mean} = (C^2 + S^2 + Z^2)^{1/2}$,

$$C = \frac{1}{m}\sum_{j=1}^{m}\left(\frac{T_j}{\sqrt{1+T_j^2}}\cos(2\theta_j)\right),$$

$$S = \frac{1}{m}\sum_{j=1}^{m}\left(\frac{T_j}{\sqrt{1+T_j^2}}\sin(2\theta_j)\right),$$

$$Z = \frac{1}{m}\sum_{j=1}^{m}\frac{SI}{\sqrt{1+T_j^2}},$$

$$SI = \begin{cases} -1 & \text{where } \varphi \geq 90^\circ \\ 1 & \text{where } \varphi < 90^\circ \end{cases},$$

$$T_j = \sqrt{\tan^{-2}(2\beta_j) + \tan^{-2}(2\gamma_j)},$$

β and γ were related to φ via the formula: $\tan^2(\varphi) = \tan^{-2}(\beta) + \tan^{-2}(\gamma)$.

2 Supplementary Figures

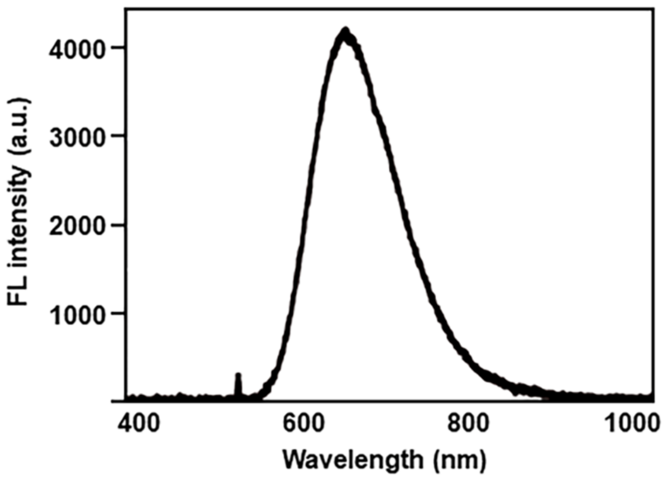

Fig. S1. The three-photon fluorescence spectrum of DCDPP-2TPA in aqueous dispersion, under the 1550 nm fs laser excitation.

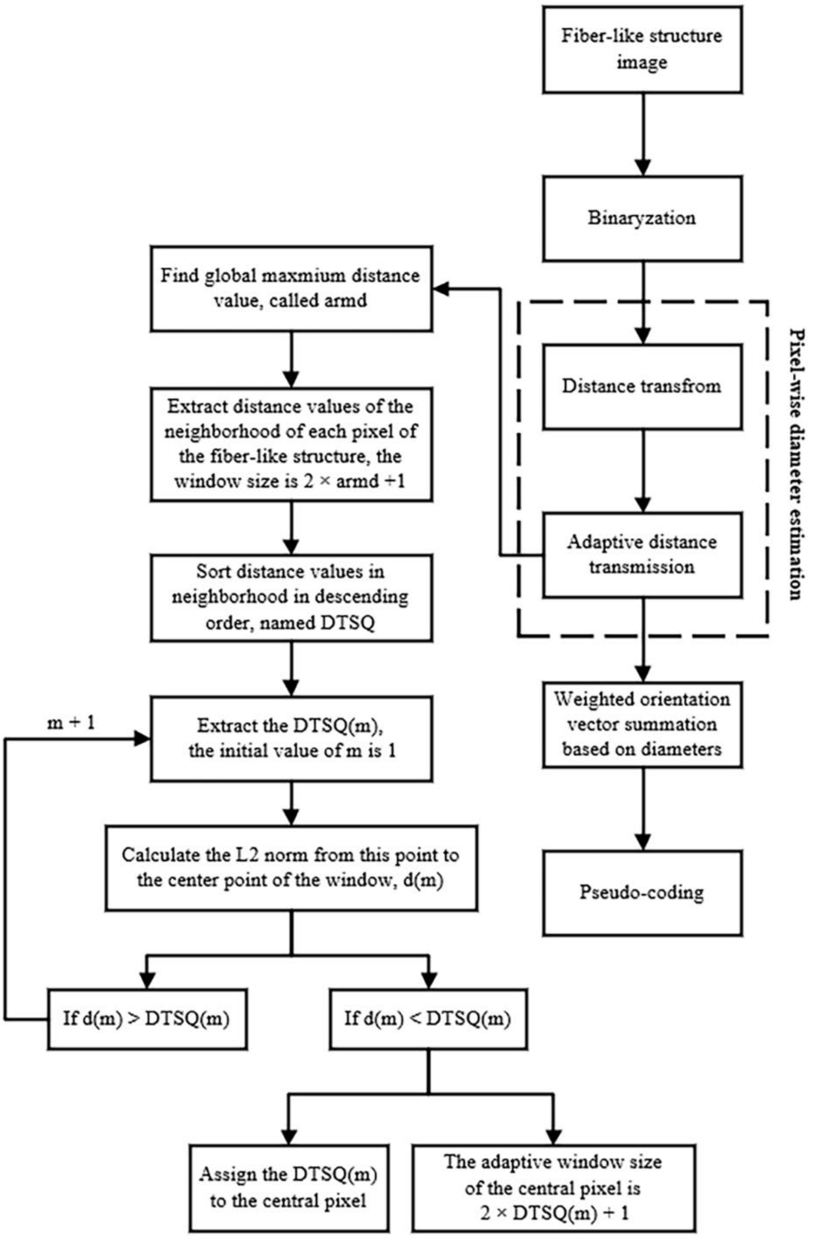

Fig. S2. Flowchart of window optimizing method.

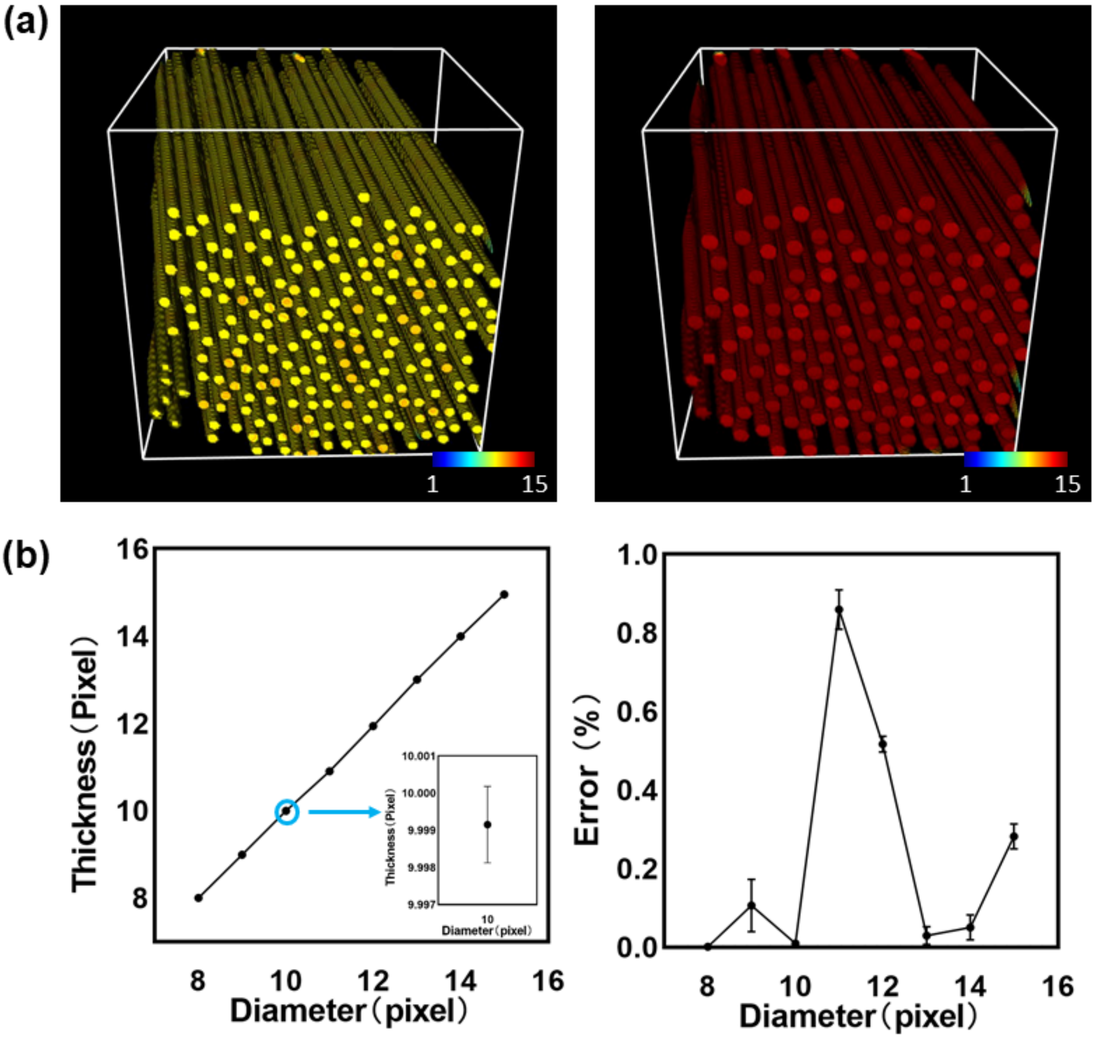

Fig. S3. Performance of the thickness algorithm. (a) Thickness maps of stacks with different fiber diameters. (b) Statistical analysis results of thickness assessment, along with the calculation error. n = 8 stacks. Analysis results demonstrate that calculated and defined thickness values follow a good linear relationship, and a superb accuracy level with the percent error less than 1% is acquired.

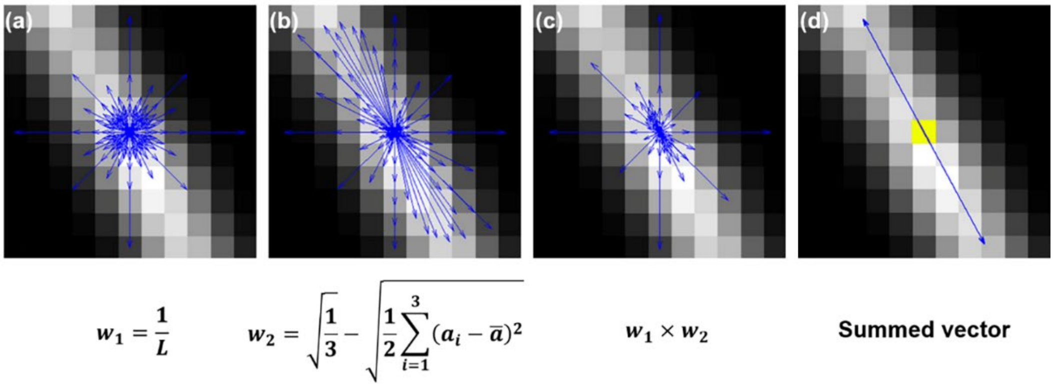

Fig. S4. Summary of weighted orientation vector summation technique for pixel-wise fiber orientation detection. (a) All the vectors through the central pixel are modified by weight factor 1 (w_1). (b) Vectors are modified by weighted factor 2 (w_2) considering the intensity variation along different directions. (c) Vectors are modified by a combination of weight factors 1 and 2. (d) The orientation of the center pixel is acquired by summation of all weighted vectors[17].

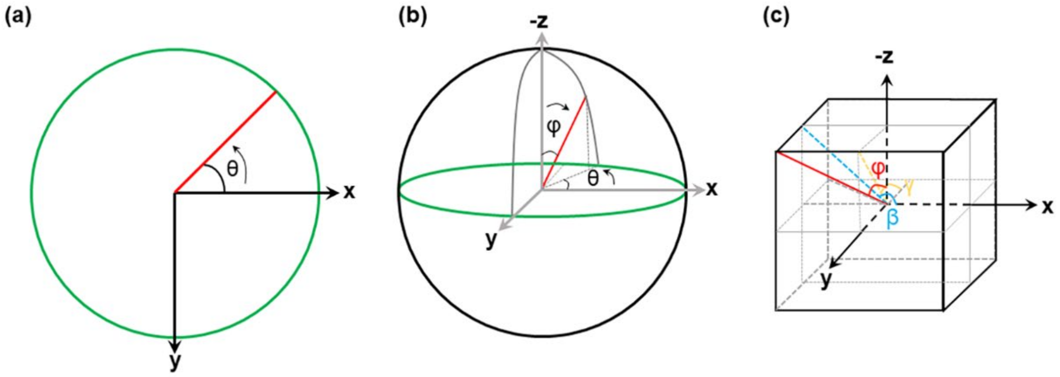

Fig. S5. Schematic of the angle definition. (a) An azimuthal angle θ is needed to describe an orientation in 2D space. (b) Both an azimuthal angle θ and a polar angle φ are needed to describe an orientation in 3D space. (c) Definition of β and γ.

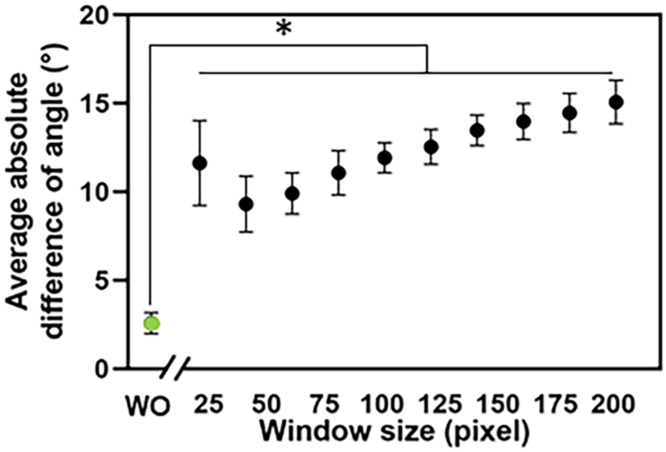

Fig. S6. Comparison in error of orientation determination between the WO method (green) and the method with fixed window (black) in 2D case. The range of fiber diameter is 1-60 pixels in each 500×500 pixels image. $n = 4$ images. *, $p < 0.05$.

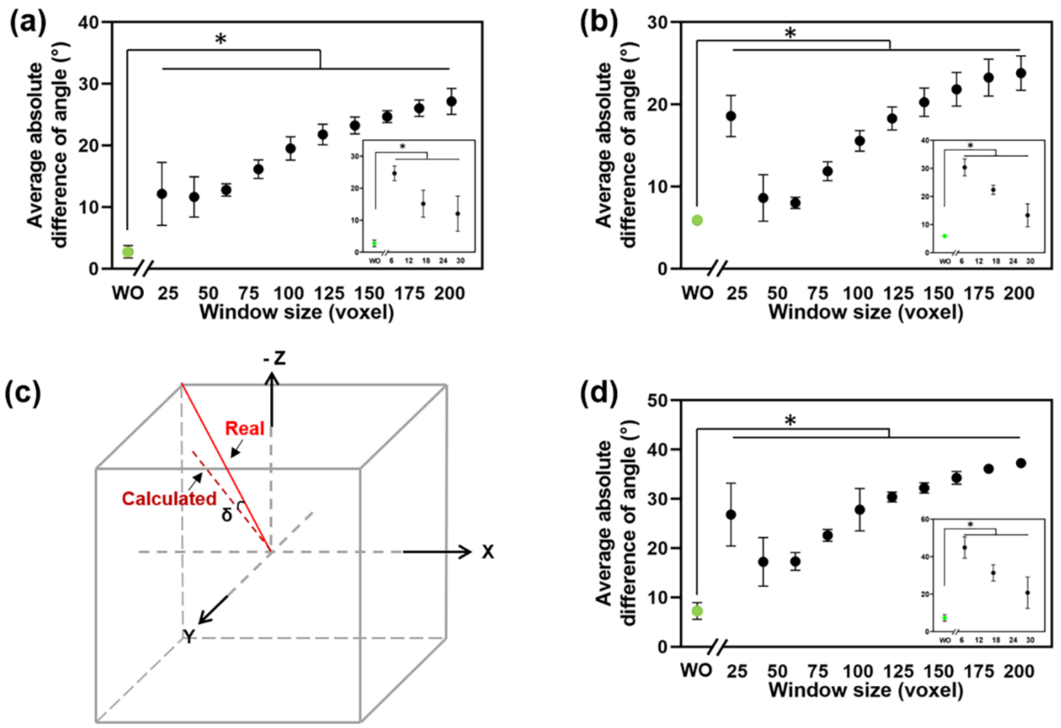

Fig. S7. Comparison in error of orientation determination between the WO method (green) and the method with fixed window (black) in 3D case. (a) θ error level. (b) φ error level. (c) Definition of the angle δ, which is the angle between the real and the calculated orientation in 3D space. (d) δ error level. The error levels acquired from window sizes at 7, 17 and 29 are shown in the inset of (a), (b) and (d). The range of fiber diameter is 8-30 voxels in each 300×300×300 voxels image. $n = 4$ images. *, $p < 0.05$.

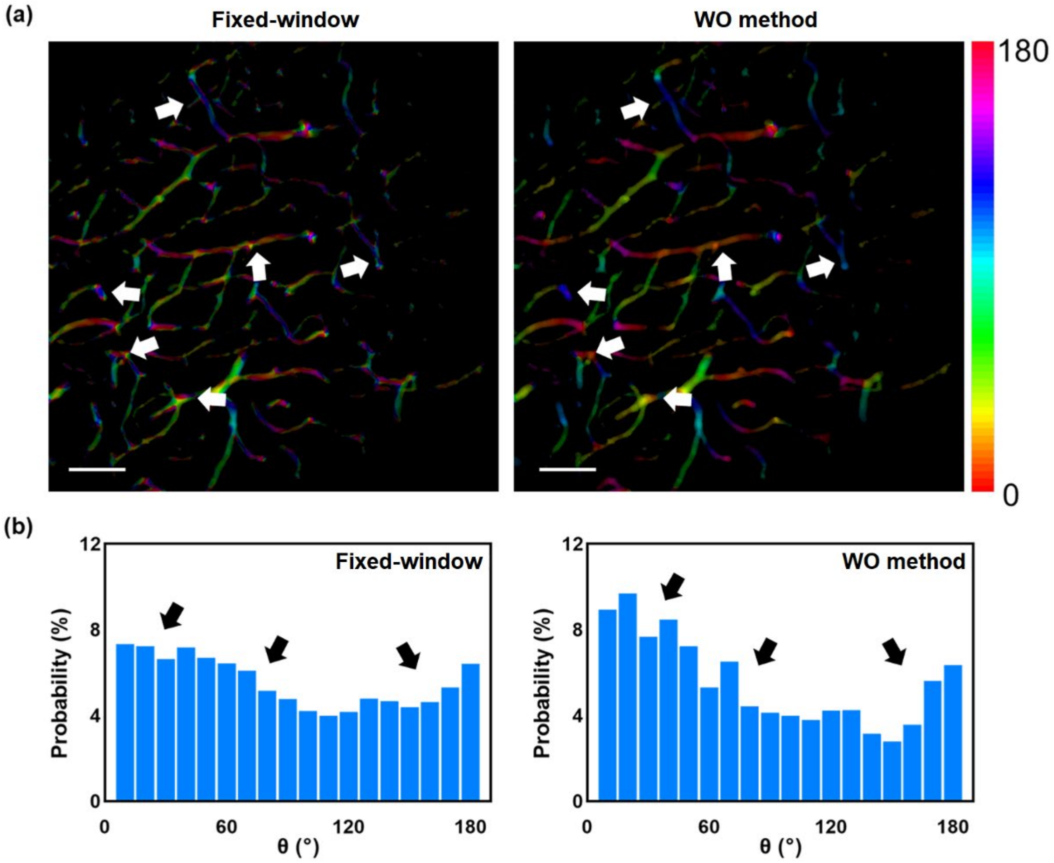

Fig. S8. Characterization of the orientation of 2D real blood vessel image based on the fixed-window method and the WO method. (a) Orientation maps from different methods, with typical regions marked out by white arrows. (b) Comparision of orientation distribution histograms from different methods, with differences marked by balck arrows. Scale bar: 50 μm.

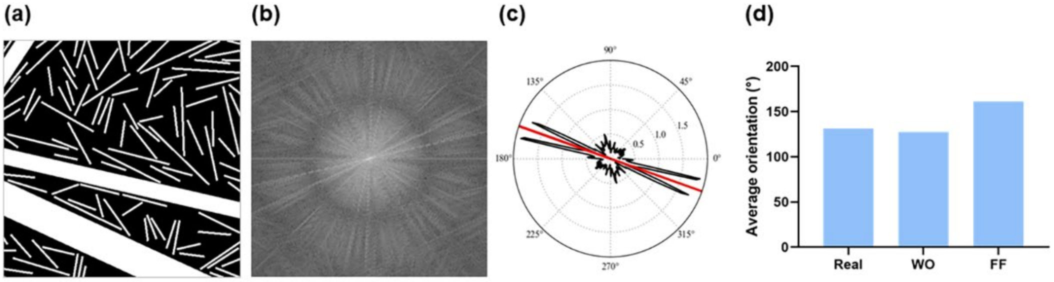

Fig. S9. Performace of the FiberFit (FF) method. (a) Simulated fiber image. (b) Fourier transform of this image. (c) Average orientation (red line in the circle) of all the fibers in the image. (d) Comparison in average orientation among ground truth (Real), WO method and FF method.

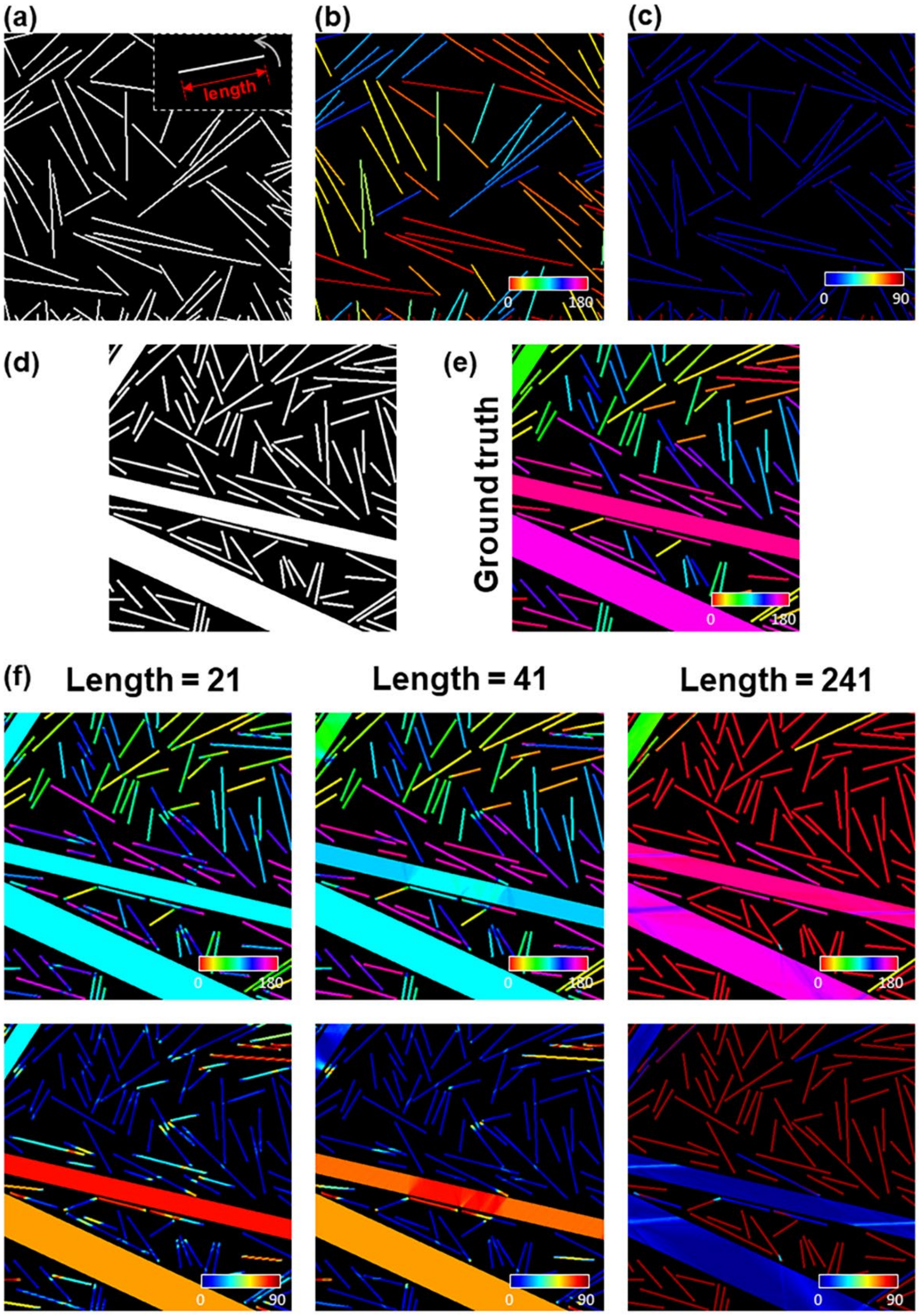

Fig. S10. Performance of the morphological method. (a) Simulated fiber image with the same diameter for all the fibers. (b) Calculated orientation map using the morphological method. (c) The error map. (d) Simulated fiber image with varying fiber diameters. (e) Ground truth of the fiber orientation. (f) The calculated fiber orientation maps using different opening lengths (top) and corresponding error maps (bottom).
